# How Does House Demolition Affect Family Conspicuous Consumption?

**DOI:** 10.3389/fpsyg.2021.741006

**Published:** 2021-10-28

**Authors:** Wei Yuan, Shuying Gong, Jun Gao

**Affiliations:** ^1^School of Business Administration, Hubei University of Economics, Wuhan, China; ^2^College of Business, Shanghai University of Finance and Economics, Shanghai, China

**Keywords:** house demolition, family conspicuous consumption, household wealth, materialism value, luxury consumption

## Abstract

Family conspicuous consumption behavior is affected by many factors. Existing pieces of literature seldom focus on the impact of house demolition on family conspicuous consumption and its underlying mechanism. Based on the mental accounting theory and conservation of resources theory, this study uses the micro-data of the 2011 China Household Finance Survey to empirically examine the relationship between house demolition and family conspicuous consumption. Robustness results suggest that house demolition positively affects household conspicuous consumption, which is not only reflected in the overall consumption level but also in the level of average consumption. Further analysis finds that household wealth and materialism value have a significant positive moderating effect on the relationship of the main effect. In addition, in order to clarify the relationship between conspicuous consumption and luxury consumption, this study finds that conspicuous consumption and luxury consumption are not completely equivalent through in-depth theoretical analysis and exploratory investigation. There are similarities in both consumption motivation and pattern, but with differences on consumer subject and object. The contribution of this research is to enrich the theory of decision-making in consumer behavior, which also has certain significance in deepening the understanding of the relationship between conspicuous consumption and luxury consumption.

## Introduction

According to official data, the total consumption of goods market in China reached 10.7 billion dollars in 2011, which accounts for a quarter of the global share, and China has become the second largest luxury consumption country in the world. By 2020, the sales of luxury goods in China still soared by 48%, reaching 53.5 billion dollars; the share of luxury consumption of China in the global market has almost doubled, and more than half of the growth of the global luxury market since 2020 has come from China (almost 65%). Therefore, China has always been a main force in the international luxury market. Veblen (1899) proposed the concept of conspicuous consumption and defined conspicuous consumption as showing off the wealth and status of oneself to others by purchasing and displaying expensive and luxurious products (Sundie et al., 2011). Therefore, behind the massive luxury consumption of Chinese consumers, it also shows the widespread phenomenon of conspicuous consumption in China.

Most of the existing pieces of literature on conspicuous consumption discuss the influence of individual motivations on conspicuous consumption, such as seeking symbolic social status and wealth signal, highlighting the identity they pursue, attracting romantic partners, and obtaining the threshold to enter the high social status circle (Sundie et al., 2011; Bellezza et al., 2017; Cannon and Rucker, 2019). However, few studies have explored the impact of external events that happen to individual consumers on the conspicuous consumption motivation of people. Our study introduces the concept of house demolition to explore the impact of house demolition on family conspicuous consumption.

Since the reform and opening up, with the development of the economy and society in China, the number and scale of house demolition have become larger. House demolition refers to the needs for national construction, urban renovation, city appearance rectification, and environmental protection; the construction unit or an individual demolishes the houses on the existing construction land, relocation, and resettlement of house owners or users, and gives relatively large amounts of economic compensation as appropriate^1^ (Wong, 2015; Cai et al., 2018; Li et al., 2019). Compared with the regular income of people, the demolition compensation is more likely to be regarded as irregular and unexpected income (Li and Xiao, 2020). Mental account theory is a theoretical framework used to explain how people evaluate and track their income through classification or labeling (mental account), and then produce different consumption tendencies according to the accounting system (Thaler, 1980, 1985; Hossain, 2018). This theory suggests that when people classify money as unexpected and windfall income in mind, they will be more inclined to use this income for hedonic consumption and spend more (Arkes et al., 1994; Hossain, 2018). Therefore, we infer that when the demolition households receive the compensation, they will have a greater willingness to choose conspicuous consumption.

One of the main purposes of conspicuous consumption is to show the status through the consumption of specific products. Early studies focus on luxury goods, high-priced products, such as luxury cars, jewelry, and other products as status signals (Veblen, 1899; Wang and Griskevicius, 2014; Ward and Dahl, 2014). In recent years, scholars have increasingly discussed some new signals that can show their identity, such as lack of leisure time, buying cool and unusual products (Warren and Campbell, 2014; Bellezza et al., 2017; Bellezza and Berger, 2020). Bellezza et al. (2017) find that a busy and high workload lifestyle is also a way for a person to show off his or her status because it represents the capable, ambitious, and indispensable status of a person. So, whether a person is really busy or not, choosing time-saving services (such as Peapod, an online grocery delivery service) and products (such as Bluetooth headsets) can also be used as signals that highlight identity. Therefore, when people choose conspicuous consumption, it may not be necessary to purchase luxury goods. This extends another important issue that is trying to inquire in this study, that is, whether conspicuous consumption and luxury consumption are equivalent. Combined with the above analysis, we suggest that, most of the existing studies regard conspicuous consumption and luxury consumption as concepts with the same connotation and do not distinguish them (Wang and Griskevicius, 2014; Garcia et al., 2019; Martinez Alfaro, 2020). However, there are still differences between conspicuous consumption and luxury consumption. For example, although the two consumption patterns are to seek the symbolic signal of status, the specific consumption choices can be different.

In summary, aiming at several research gaps in conspicuous consumption, we carry out two studies. Study 1 is a large sample empirical analysis based on the survey data China Household Finance Survey, which mainly discusses two problems: first, the impact of house demolition on conspicuous consumption; second, it further studies the boundary effect of house demolition on conspicuous consumption, that is, to explore whether the level of household wealth and materialism value moderates the main effect. What is more, we construct the robustness test with four different methods to ensure the robustness of the research results. Study 2 is an exploratory study based on the theoretical deduction of the connotation of conspicuous consumption and luxury consumption; we analyze the similarities and differences between them by using an exploratory questionnaire survey to clarify the relationship. A series of new research findings on conspicuous consumption in our study provides positive theoretical and practical enlightenment for consumers, enterprises, and the government.

## Study 1: The Effect of House Demolition on Family Conspicuous Consumption

### Theoretical Analysis and Research Hypothesis

#### House Demolition and Family Conspicuous Consumption

Veblen (1899) proposes the concept of conspicuous consumption, which describes that people achieve the purpose of highlighting their social status by obtaining and displaying expensive and luxury products. Through conspicuous consumption, people will feel their power and social self-identification improvement (Cannon and Rucker, 2019). Therefore, attracting the attention of others and showing their identity, power, and image to others are the main motivations for people to choose conspicuous consumption (Belk, 1988; Rucker and Galinsky, 2008; Lee and Shrum, 2012). In addition, relevant studies also find that luxury consumption of people may also meet many motivations, such as attracting the partner(s), entering a high-level social circle, reflecting the comparative advantage with competitors (Griskevicius et al., 2007; Rivera, 2010; Sundie et al., 2011; Wang and Griskevicius, 2014; Bellezza et al., 2017). According to the above research, people choose conspicuous consumption mostly for hedonic motivation. Therefore, we believe that conspicuous consumption can well meet the hedonic needs of people.

According to mental account theory, people will use a series of cognitive labels or mental accounts to track and evaluate income and resources, and these accounts further influence their consumption attitudes and behaviors (Kahneman and Tversky, 1984; Thaler, 1985; Vana et al., 2018). Consumers usually classify their money according to the scenario of income they received and choose a matching consumption mode according to the source of wealth (Levav and McGraw, 2009; Reinholtz et al., 2015). Among many classification labels, a group of classification labels that is often explored in previous studies divides income into regular income and windfall income according to the source of income. Regular income is the part of the income from individual labor, which is “hard-won” and is actually expected income. In contrast, windfall income is individual non-labor income, which is closer to “getting without work” and is really unexpected (Kivetz, 1999). There are significant differences in calculation rules between two different mental accounts (Thaler, 1985). The calculation rule of regular income is “utilitarian processing,” which focuses more on “utility” in the calculation process and pursues the maximization of rational cognitive utility. However, the calculation rule of windfall income is “hedonistic processing,” which pays more attention to “emotion” in the calculation process and pursues the maximization of emotional satisfaction (Li et al., 2007). According to the consumers' classification of income from the source, consumers tend to use regular income (such as wage income) to buy daily necessities and use windfall income (such as lottery income) for hedonic consumption and spend more on this part of the income (Hossain, 2018).

For consumers, obtaining relatively large economic compensation due to house demolition is a kind of irregular and unexpected income (Li and Xiao, 2020). As a result, consumers tend to classify this income as windfall income and have a higher willingness to spend more windfall income, which, in turn, drives them to pursue hedonic consumption such as conspicuous consumption. Thus, we infer that, compared with non-demolished households, demolished households may be more prone to conspicuous consumption due to windfall income of demolition compensation, that is, house demolitions have a positive effect on conspicuous consumption. In addition, we believe that the stimulation of house demolition on the level of conspicuous consumption is not only reflected in the overall consumption level but also in the average consumption level. So, here are the following two hypotheses:

***H1: House demolition significantly improves the overall level of family*** ***conspicuous consumption;***
***H2: House demolition significantly increases the average level of family*** ***conspicuous consumption*.**

#### House Demolition Household Wealth and Family Conspicuous Consumption

As discussed above, a mental account of people for the division of money will affect their consumption attitude toward the money. There is no doubt that families with different economic conditions will naturally affect how people classify and label money. Therefore, we suggest that household wealth will play a moderator role in the relationship between house demolition and family conspicuous consumption. Based on the resource conservation theory, which suggests that the behavior of people is a function of resources, they will try to reserve, protect, and obtain resources that are valuable, and also prevent potential and actual loss of resources by investing resources (Hobfoll, 1989, 2011; Xu et al., 2021). Valuable resources include money resources, physical resources, motivation resources, social resources, et al. (Wang et al., 2011; Gao et al., 2013; Zhu et al., 2017). According to the differences in the perceived importance of resources, people will try their best to reduce the loss of resources in the threat situation and invest abundant resources to further obtain additional resources (Hobfoll, 2011; Ye et al., 2019).

Therefore, we expect that demolished households with a high-wealth level will have a stronger incentive to spend the extra income on conspicuous consumption than demolished households with a low-wealth level. Relatively large economic compensation due to house demolition will be an important wealth resource for households. For households with a high-wealth level, the importance of this windfall income is much low than that of households with a low-wealth level. Therefore, households with a low-wealth level will pay more attention to economic compensation due to house demolition, since it is an important resource and can more effectively improve the overall quality of life from many aspects. According to the resource conservation theory, when people have more abundant resources, they are less susceptible to the loss of this part of resources and more likely to invest this part of resources to obtain additional resources (Hobfoll, 2011; David et al., 2021). Therefore, households with a low-wealth level will regard economic compensation due to house demolition as valuable resources to a higher extent they will spend relatively less money to avoid psychological pressure and loss caused by resource loss. For those with a high-wealth level, they have relatively rich wealth resources, so they will have a stronger willingness to invest this part of resources, such as conspicuous consumption. In addition, due to the relatively high-wealth level, it has little impact on them to use this part of economic compensation from house demolition for consumption, and the corresponding sense of loss is weak. Based on the above analysis, we make the following inferior: the household wealth level will moderate the impact of house demolition on family conspicuous consumption. Specifically, for a household with a high-wealth level, house demolition has a stronger positive impact on conspicuous consumption; conversely, the above effect is weaker for households with a low-wealth level. Therefore, we propose Hypothesis 3 and Hypothesis 4:

***H3: The level of household wealth moderates the main effect between house*** ***demolition and the overall level of family conspicuous consumption. In*** ***specific, a high level of household wealth (vs. a low level of household wealth)*** ***significantly increases the overall level of conspicuous consumption of the*** ***demolished household; a low level of household wealth (vs. a high level of*** ***household wealth) significantly decreases the overall level of conspicuous*** ***consumption of non-demolished households*.**
***H4: The level of household wealth moderates the main effect between house*** ***demolition and the average level of family conspicuous consumption. In*** ***specific, a high level of household wealth (vs. a low level of household wealth)*** ***significantly increases the average level of conspicuous consumption of the*** ***demolished household; a low level of household wealth (vs. a high level of*** ***household wealth) significantly decreases the average level of conspicuous*** ***consumption of non-demolished households*.**

#### House Demolition, Materialism Value, and Family Conspicuous Consumption

In addition to the level of household wealth, we believe that individual materialism value is also an important moderator variable affecting the relationship between house demolition and family conspicuous consumption. Fournier and Richins (1991) define materialism value as a value orientation, which highly emphasizes the realization of important goals in life through acquisition or possession. Richins and Dawson (1992) further summarize three dimensions of materialism value, possession and acquisition are the core concerns of life, obtaining more quantity and more types of things will increase happiness, and possession is the definition of success. Although different scholars have differences in the specific definition of materialism value (Csikszentmihalyi and Halton, 1981; Belk, 1985; Kasser and Ryan, 1996; Yoo et al., 2021), it has gradually derived that scholars explore materialism value from the perspective of individualism and culture (Belk, 2015). In recent years, scholars have even discussed the perception of materialism value in the consumption of virtual products, such as digital products (Atanasova and Eckhardt, 2021). However, scholars generally believe that materialism value is a kind of consumption logic in which people show their identity, establish images, pursue happiness, and obtain self-value by possessing objects and acquiring experiences (Belk, 2015; Huang et al., 2018; Atanasova and Eckhardt, 2021).

In view of the connotation of materialism value, we believe that materialism value will further enhance the willingness of demolition households to use economic compensation from demolition for conspicuous consumption. On the one hand, consumers with materialism tendencies will regard possession and acquisition as the means to obtain happiness and the standard of happiness in life and will have a stronger willingness to increase the quantity and types of goods (Richins, 2017). Conspicuous consumption is an effective way for people through consumption to show status and increase their sense of happiness and worth. Therefore, conspicuous consumption is an ideal choice for consumers with materialism value, and these consumers will have a stronger willingness to use economic compensation for conspicuous consumption to improve their happiness. On the other hand, people with materialistic values are more likely to make impulsive purchases, preferring to sacrifice long-term benefits for immediate pleasure (Yoon and Hyeongmin, 2016; Huang et al., 2018). Therefore, compared with consumers with non-materialistic values, when consumers with materialistic value receive economic compensation from house demolition, they will have a stronger willingness to use the money to increase consumption for happiness, which also promotes their choice of conspicuous consumption. To sum up, we infer that materialism value will moderate the effect of house demolition on family conspicuous consumption. Specifically, for demolition households with materialism value, house demolition has a stronger positive impact on family conspicuous consumption; conversely, the above effect was weaker for demolition households with non-materialism value. Based on this judgment, we propose Hypotheses 5 and 6:

***H5: Materialism value significantly moderates the main effect between house*** ***demolition and the overall level of family conspicuous consumption. In*** ***specific, materialism value (vs. non-materialism value) significantly increases*** ***the overall level of conspicuous consumption of demolished households;*** ***non-materialism value (vs. materialism value) significantly decreases the*** ***overall level of conspicuous consumption of non-demolished households*.**
***H6: Materialism value significantly moderates the main effect between house*** ***demolition and the average level of family conspicuous consumption. In*** ***specific, materialism value (vs. non-materialism value) significantly increases*** ***the average level of conspicuous consumption of demolished households;*** ***non-materialism value (vs. materialism value) significantly decreases the*** ***average level of conspicuous consumption of non-demolished households*.**

### Research Design

#### Data

We used the China Household Finance Survey (CHFS) data in 2011. CHFS (2011) is a survey organized by the Survey and Research Center for China Household Finance of Southwestern University of Finance and Economics. It interviews respondents by random sampling, covers respondents in 25 provinces (autonomous regions and municipalities), 80 counties, and 320 communities. We extracted information about family conspicuous consumption, household characteristics, and the area where the household is located from the survey. Finally, we obtained more than 6,000 samples.

#### Variable Definition

There is still not a consensus about the definition of conspicuous consumption of the explained variable in academics. However, these different opinions can be roughly divided into two categories. The first type of viewpoint is represented by Veblen, who believes that conspicuous consumption is the exclusive consumption mode of the “leisure class” (Veblen, 1899). However, with the rapid development of society and the deepening of academics, more and more scholars have gradually adopted the view that “conspicuous consumption is a universal mode of consumption” (Zhu, 2001; Charles et al., 2009). Taking China as the research background, we support the second view that conspicuous consumption is applicable to consumers of all classes combined with the phenomenon of conspicuous consumption in daily life. Similarly, our study supports the following point of view: the scope of conspicuous consumption is not limited to luxury goods or expensive goods, and consumers can spend money on both high-price and low-price goods to achieve conspicuous purposes (Bagwell and Bernheim, 1996). Since conspicuous consumption must be a kind of visible consumption (Charles et al., 2009), conspicuous consumption of people can rely on visible ordinary clothes, shoes, and other non-expensive commodities in life (Frank, 1985). In view of the above, referring to the existing literature (Charles et al., 2009; Meng et al., 2010), we used the total consumption of the family buying clothes to measure the family conspicuous consumption. The specific definition methods and corresponding data processing methods are as follows:

##### Dependent Variables

The *overall level of family conspicuous consumption* and the *average level of family conspicuous consumption* are two dependent variables set in this study. First, we used the total household consumption of clothes purchased in the CHFS (2011) questionnaire survey (unit: 1,000 RMB) to measure the overall level of family conspicuous consumption. Among them, the total family consumption for buying clothes mainly includes “consumer expenditures for buying clothes for themselves,” “consumer expenditures for buying clothes for spouses,” and “consumer expenditures for buying clothes for children.” Then, we used the ratio of the total consumption of clothes purchased by the family to the number of family members to measure the average level of family conspicuous consumption.

##### Core Independent Variable

The core independent variable select in our study is *house demolition*. Referring to the existing literature (Chai, 2014; Yuan and Huang, 2018), we defined the families who have experienced house demolition in the CHFS (2011) questionnaire as the demolished households and assigned them the value of 1. On the contrary, families who have not experienced house demolition are defined as non-demolished households and assigned the value of 0.

##### Moderator Variables

The level of *household wealth* and the *materialism value* are the two moderator variables in our study. We adopted the most commonly used method in current academic research to define the household wealth, that is using the average value of the whole household's wealth as the criterion. If the household wealth is higher than the average, it is regarded as a household with high-level wealth and assigned the value 1; if the family wealth value is lower than the average, it is regarded as a household with low-level wealth and assigned the value of 0. Among them, household wealth mainly includes two parts: financial assets and non-financial assets.

*Materialism Value*. We use the items in CHFS (2011) questionnaire, “When your family assets rise, will you spend more money?” 1 means high willing, willing, and general; 0 means unwilling and quite unwilling. Thus, 1 is also defined as a household (family) with materialism value; in contrast, 0 is defined as a household (family) with non-materialism value.

##### Control Variables

Besides variables discussed above and in prior literature, we mainly set the following control variables: (1) Household income. Household income mainly includes wage income, transfer income, and other income. We added 1 to the household income and then took the natural logarithm; (2) the number of houses. The number of houses mainly refers to the specific number of houses with property rights. If the family does not own a house with property rights, then it is assigned the value of 0; (3) gender of the head of household. If the head of the household is male, then we assigned the value of 1. If the head of the household is female, then we assigned the value of 0; (4) age of the head of a household. The age of the head of the household mainly refers to the actual age of the head of the household; (5) the education level of the head of the household. The education level of the head of the household mainly refers to the education years received by him or her. Among them, 0 stands for never going to school, 6 stands for elementary school, 9 stands for junior high school, 12 stands for high school, 13 stands for technical secondary school or vocational high school, 15 stands for college or higher vocational school, 16 stands for university undergraduate, 19 stands for master degree, and 22 stands for doctoral degree; (6) marital status. Marital status mainly refers to the marital status of the head of the household. If the marriage status is “married,” “divorced,” or “widowed,” it is defined as “married” and assigned a value of 1. If the marriage status is “unmarried” or “cohabiting together,” it is defined as “unmarried” and assigned a value of 0; (7) health situation: We used the item in CHFS (2011) questionnaire to measure a health situation; the item is “Compared with your peers, how is your physical condition?”; 1 means very poor, 2 means poor, 3 means general, 4 means good, and 5 means very good; (8) Social insurance: In Lin et al.'s (2017) study, if a family has at least any one of social pension insurances, retirement wages, corporate annuities, social medical insurances, unemployment insurances, or housing provident funds, it is regarded as a family with social insurance and is assigned the value of 1; otherwise, it will be regarded as a family that does not have social insurance and assigned the value of 0.

#### Model Setting

In order to investigate the relationship between house demolition and the overall level of family conspicuous consumption, we constructed the following model on the basis of controlling the household characteristics and the family characteristics:


(1) $$\begin{array}{r} {Con\text{\_}all}_{i}=\beta_{0}+\beta_{1}Dem_{i}+\Gamma X_{i}+\varepsilon \end{array}$$

where *Con*_*all*_*i*_ indicates the overall level of family conspicuous consumption, which is a continuous variable; the core independent variable *Dem*_*i*_ is a dummy variable for house demolition; and *X*_*i*_ captures the control variables; β_0_and β_1_ are the regression coefficients, Γ is the corresponding regression coefficient matrix; *i* indicates each household; in addition, ε represents the error term.

In order to investigate the relationship between house demolition and the average level of family conspicuous consumption, we constructed the following model (2) based on controlling the household characteristics and the family characteristics:


(2) $$\begin{array}{r} {Con\text{\_}average}_{i}=\beta_{0}+\beta_{1}Dem_{i}+\Gamma X_{i}+\varepsilon \end{array}$$

Among them, *Con*_*average*_*i*_ is the average level of family conspicuous consumption, which is also a continuous variable; *Dem*_*i*_ and *X*_*i*_ are set as the model (1).

In order to analyze whether the household wealth and materialism value significantly moderate the main effect between house demolition and family conspicuous consumption, we included the interaction between house demolition and household wealth, and the interaction between house demolition and materialism value in the model (3) and model (4) to avoid the problem of multicollinearity; we centralized the three variables: house demolition, household wealth, and materialism value.


(3) $$\begin{array}{r} {Con\text{\_}all}_{i}=\beta_{0}+\beta_{1}Dem\text{\_}z_{i}+\beta_{2}{Wea\text{\_}z}_{i}+\beta_{3}Dem\text{\_}z_{i}\times \\ {Wea\text{\_}z}_{i}+\beta_{4}{Mat\text{\_}z}_{i}+\;\beta_{5}Dem\text{\_}z_{i}\times {Mat\text{\_}z}_{i}++\Gamma X_{i}+\varepsilon \end{array}$$

(4) $$\begin{array}{r} {Con\text{\_}average}_{i}=\beta_{0}+\beta_{1}Dem\text{\_}z_{i}+\beta_{2}{Wea\text{\_}z}_{i}+\beta_{3}Dem\text{\_}z_{i}\times \\ {Wea\text{\_}z}_{i}+\beta_{4}{Mat\text{\_}z}_{i}+\beta_{5}Dem\text{\_}z_{i}\times {Mat\text{\_}z}_{i}++\Gamma X_{i}+\varepsilon \end{array}$$

*Dem*_*z*_*i*_indicates the house demolition after centralized processing, *Wea*_*z*_*i*_ indicates the household wealth after centralized processing, and *Mat*_*z*_*i*_ indicates the materialism value after centralized processing. The setting of other variables is the same as the model (1).

#### Descriptive Statistics and Correlation Analysis

Table 1 shows the results of the descriptive statistics of the main variables. The results show that the average of the overall family conspicuous consumption level is 3.079, the average of the family conspicuous consumption level is 0.868, and the average of house demolition is 0.111. The mean of the interaction between house demolition and materialism value is −0.004, and the mean of the interaction between house demolition and household wealth is −0.002. From the results of the correlation analysis in Table 1, house demolition is significantly positively correlated with the overall level of family conspicuous consumption (*p* < 0.05) and the average level of family conspicuous consumption (*p* < 0.05); the interaction between house demolition and materialism value is significantly positively correlated with the overall level of family conspicuous consumption (*p* < 0.01), and the average level of family conspicuous consumption (*p* < 0.01); the interaction between house demolition and a household wealth level is significantly positively correlated with the overall level of family conspicuous consumption (*p* < 0.1), and the average family conspicuous consumption (*p* < 0.05). Further tests will analyze the relationship between the above variables in depth.

**Table 1:** Descriptive statistics and correlation analysis.

**Variable**	**Number**	**1**	**2**	**3**	**4**	**5**	**6**	**7**	**8**
The overall level of family conspicuous consumption	1	1							
Average level of family conspicuous consumption	2	0.985^***^	1						
House demolition	3	0.034^**^	0.040^**^	1					
Interaction 1	4	0.049^***^	0.054^***^	−0.073^***^	1				
Interaction 2	5	0.031^*^	0.036^**^	−0.031^**^	0.110^***^	1			
Materialism value	6	0.089^***^	0.082^***^	−0.031^**^	0.034^***^	0.001	1		
Household wealth	7	0.229^***^	0.208^***^	−0.013	0.001	−0.003	0.114^***^	1	
Male	8	−0.067^***^	−0.074^***^	−0.023^*^	0.002	0.015	0.021^*^	0.094^***^	1
Age	9	−0.154^***^	−0.158^***^	0.066^***^	−0.003	−0.009	−0.197^***^	−0.157^***^	0.090^***^
Education level	10	0.266^***^	0.270^***^	0.025^*^	−0.015	−0.005	0.164^***^	0.258^***^	−0.001
Married	11	0.015	0.014	−0.003	0.006	−0.017	−0.040^***^	−0.029^**^	−0.014
Number of family members	12	−0.137^***^	−0.207^***^	−0.047^***^	0.010	0.013	0.051^***^	0.098^***^	0.132^***^
Number of houses	13	0.123^***^	0.108^***^	0.017	0.008	0.038^***^	0.041^***^	0.207^***^	0.070^***^
Household income	14	0.297^***^	0.298^***^	−0.028^**^	−0.004	−0.018	0.132^***^	0.213^***^	0.042^***^
Health situation	15	0.080^***^	0.074^***^	−0.018	−0.023^*^	−0.028^**^	0.090^***^	0.174^***^	0.070^***^
Social insurance	16	0.021	0.017	0.051^***^	0.012	−0.008	−0.005	0.040^***^	0.020
Mean		3.079	0.868	0.111	−0.004	−0.002	0.751	0.434	0.666
Standard deviation		5.839	1.854	0.314	0.142	0.155	0.432	0.496	0.472
Observation		3,331	3,331	6,132	6,132	6,132	6,134	6,134	6,133
**Variable**	**Number**	**9**	**10**	**11**	**12**	**13**	**14**	**15**	**16**
Age	9	1							
Education level	10	−0.412^***^	1						
Married	11	0.311^***^	−0.167^***^	1					
Number of family members	12	−0.094^***^	−0.096^***^	0.189^***^	1				
Number of houses	13	−0.001	0.060^***^	0.086^***^	0.156^***^	1			
Household income	14	−0.366^***^	0.411^***^	−0.134^***^	−0.092^***^	0.066^***^	1		
Health situation	15	−0.229^***^	0.177^***^	−0.087^***^	−0.015^***^	0.037^***^	0.173^***^	1	
Social insurance	16	0.142^***^	0.028^**^	0.127^***^	0.026^*^	0.065^***^	0.011	−0.051^***^	1
Mean		50.146	9.424	0.947	3.340	1.063	0.513	3.411	0.940
Standard deviation		14.487	4.328	0.224	1.558	0.576	0.696	0.945	0.238
Observation		6,133	6,072	6,064	6,133	6,134	6,133	5,152	5,117

*Interaction 1 refers to the centralized interaction between house demolition and materialism value, and Interaction 2 refers to the centralized interaction between house demolition and household wealth*.

* * p <0.1*;

** * p <0.05*;

*** * p <0.01*.

### Empirical Results

#### The Main Effects

Table 2 shows the regression results between house demolition and family conspicuous consumption. Model 1 and Model 5 are basic models both including core independent variables, moderator variables, and control variables at the overall level and the average level, respectively. Model 2 and Model 3 examine the moderating effect of household wealth and materialism value on the main effect between house demolition and family conspicuous consumption at the overall level. Model 6 and Model 7 test the moderating effect of household wealth and materialism value on the main effect between house demolition and household conspicuous consumption at the average level. Model 4 and Model 8 are the full models with all variables at the overall level and the average level, each model has significant explanatory power.

**Table 2:** Results of house demolition on family conspicuous consumption.

**Dependent variable**	**The overall level of family conspicuous consumption**				**The average level of family conspicuous consumption**			
**Model**	**(1)**	**(2)**	**(3)**	**(4)**	**(5)**	**(6)**	**(7)**	**(8)**
House demolition	0.977^***^	0.829^**^	1.108^***^	0.938^**^	0.316^***^	0.264^**^	0.361^***^	0.302^**^
	(0.371)	(0.378)	(0.371)	(0.378)	(0.118)	(0.120)	(0.118)	(0.120)
House demolition × household wealth		1.457^**^		1.749^**^		0.503^**^		0.604^**^
		(0.739)		(0.740)		(0.235)		(0.235)
House demolition × materialism value			3.533^***^	3.736^***^			1.220^***^	1.291^***^
			(0.900)	(0.904)			(0.287)	(0.288)
Male	−0.976^***^	−0.967^***^	−0.982^***^	−0.971^***^	−0.306^***^	−0.303^***^	−0.308^***^	−0.304^***^
	(0.245)	(0.245)	(0.245)	(0.245)	(0.078)	(0.078)	(0.078)	(0.078)
Age	−0.007	−0.007	−0.009	−0.008	−0.001	−0.001	−0.001	−0.001
	(0.011)	(0.011)	(0.011)	(0.011)	(0.004)	(0.004)	(0.004)	(0.004)
Education level	0.166^***^	0.168^***^	0.167^***^	0.170^***^	0.048^***^	0.049^***^	0.049^***^	0.049^***^
	(0.032)	(0.032)	(0.032)	(0.032)	(0.010)	(0.010)	(0.010)	(0.010)
Married	1.751	1.751	1.663	1.658	0.511	0.511	0.481	0.479
	(3.966)	(3.964)	(3.956)	(3.952)	(1.262)	(1.262)	(1.259)	(1.258)
Number of family members	−0.195^**^	−0.194^**^	−0.192^**^	−0.192^**^	−0.174^***^	−0.174^***^	−0.173^***^	−0.173^***^
	(0.088)	(0.088)	(0.088)	(0.088)	(0.028)	(0.028)	(0.028)	(0.028)
Number of houses	0.792^***^	0.777^***^	0.801^***^	0.783^***^	0.223^***^	0.218^***^	0.226^***^	0.220^***^
	(0.185)	(0.185)	(0.185)	(0.185)	(0.059)	(0.059)	(0.059)	(0.059)
Family income	1.575^***^	1.574^***^	1.559^***^	1.557^***^	0.491^***^	0.491^***^	0.485^***^	0.485^***^
	(0.173)	(0.173)	(0.173)	(0.172)	(0.055)	(0.055)	(0.055)	(0.055)
Household wealth	1.720^***^	1.749^***^	1.722^***^	1.757^***^	0.494^***^	0.504^***^	0.495^***^	0.507^***^
	(0.223)	(0.224)	(0.223)	(0.223)	(0.071)	(0.071)	(0.071)	(0.071)
Materialism value	0.290	0.287	0.225	0.218	0.073	0.072	0.050	0.048
	(0.267)	(0.267)	(0.267)	(0.267)	(0.085)	(0.085)	(0.085)	(0.085)
Health situation	0.040	0.043	0.029	0.032	0.003	0.004	−0.001	0.001
	(0.120)	(0.120)	(0.120)	(0.120)	(0.038)	(0.038)	(0.038)	(0.038)
Social insurance	−0.011	−0.004	0.044	0.056	−0.018	−0.016	0.001	0.005
	(0.462)	(0.461)	(0.461)	(0.460)	(0.147)	(0.147)	(0.147)	(0.146)
*F*-value	38.78^***^	36.14^***^	37.17^***^	34.97^***^	40.50^***^	37.79^***^	39.01^***^	36.77^***^
*R* ^2^	0.1432	0.1444	0.1479	0.1496	0.1486	0.1500	0.1542	0.1561
Observations	2,797	2,797	2,797	2,797	2,797	2,797	2,797	2,797

* * p <0.1*;

** * p <0.05*;

*** * p <0.01*.

*The values in parentheses are standard errors*.

The result of Model 1 shows that there is a significant positive correlation between house demolition and the overall level of family conspicuous consumption (beta = 0.977, *p* < 0.01). The result is still robust in subsequent Model 2, Model 3, and Model 4. Therefore, H1 is supported, that is, house demolition significantly increases the overall level of family conspicuous consumption. The result of Model 5 shows that there is a significant positive correlation between house demolition and the average level of family conspicuous consumption (beta = 0.316, *p* < 0.01). This result is still robust in subsequent Model 6, Model 7, and Model 8. So, H2 is also supported, that is, house demolition significantly increases the average level of family conspicuous consumption.

#### The Moderating Effects

The result of Model 2 shows that the interaction between house demolition and household wealth is significantly positively correlated with the family's overall conspicuous consumption (beta = 1.457, *p* < 0.05). This result is still robust in the full Model 4. Therefore, H3 is supported, that is, the level of household wealth plays a significant positive role in moderating the relationship between house demolition and the overall level of family conspicuous consumption. The result of Model 3 shows that the interaction between house demolition and materialism value is significantly positively correlated with the overall level of family conspicuous consumption (beta = 3.533, *p* < 0.01). This result is still robust in the full Model 4. Therefore, Hypothesis 5 is supported, that is, materialism value plays a significant positive role in moderating the relationship between house demolition and the overall level of family conspicuous consumption. The result of Model 6 shows that the interaction between house demolition and household wealth is significantly positively correlated with the average family conspicuous consumption (beta = 0.503, *p* < 0.05). This result is still robust in the full Model 8. Therefore, H4 is supported, that is, the level of household wealth has a significant positive effect on the relationship between house demolition and the average level of family conspicuous consumption. The result of Model 7 shows that the interaction term between house demolition and materialism value is significantly positively correlated with the average family conspicuous consumption (beta = 1.220, *p* < 0.01). This result is still robust in the full Model 8. Therefore, H6 is supported, that is, materialism value plays a significant positive role in moderating the relationship between house demolition and the average level of family conspicuous consumption. In sum, we verified that the level of household wealth and materialism value plays significant moderating roles in the main effect between house demolition and family conspicuous consumption from the overall and average levels, respectively.

### Robustness Checks

#### Endogenous Test

The previous part has discussed and confirmed that house demolition significantly affects family conspicuous consumption behavior. However, the level of family conspicuous consumption may also affect the decision of family house demolition. Specifically, whether a family needs to rely on house demolition to obtain compensation for supporting the family conspicuous consumption behavior, to a certain extent, determines the house demolition decision of the family. If necessary, the family may choose to obey the demolition plan of the government department and become a demolished household; on the contrary, if it is not necessary, the family may refuse to obey the demolition plan of the government department and become a non-demolished household. It can be seen that there may be a reverse causality relationship between house demolition and family conspicuous consumption.

However, reverse causality may lead to an endogenous problem, which may lead to the bias of the results. In order to alleviate this problem, we referred to the method of Yuan and Huang (2018) and used the number of households to be demolished in each region as an instrumental variable for house demolition. For the number of demolished households in each region, we used the number of demolished households in each province in the CHFS (2011) survey data, plus 1, and then took the natural logarithm to measure. The reason for using the number of demolished households in each region as an instrumental variable for house demolition are: on the one hand, whether households in each region experience house demolition or not had a decisive effect on the number of demolished households in the region, indicating that house demolition and the number of demolished households in each region are highly related. On the other hand, factors related to individual or family characteristics, such as the years of education, gender, family income, and family assets, may directly affect the level of family conspicuous consumption. However, the number of households to be demolished in each region, as a nonindividual or family characteristic factor at the provincial and municipal levels, may not directly affect the family conspicuous consumption. It can be seen that the number of households to be demolished in each region meets the two requirements of the instrument variable. In addition, through the weak tool identification test and the exogenous test, we confirmed that the number of households to be demolished in each region is an effective and strong instrument variable.

#### Modify the Model Setting

Based on the empirical evidence of the existing literature and the principle of data availability, we controlled a number of variables related to individual and family characteristics in the model setting. However, in order to avoid bias in research results due to the selection of control variables, we added additional control variables to the original model settings for the robustness test. Considering the fact that ethnicity, whether a Communist Party of China (CPC) member, household debt status, and urban dummy variables may all affect household consumption decisions. In this regard, the four variables of “ethnicity,” “whether they are members of the Communist Party of China, “household debt,” and “urban dummy variables” are used as new control variables in the model. Among them, for the ethnicity, we used 1 to represent the Han ethnicity and 0 to represent others; for whether a member of the Communist Party of China, we used 1 to represent a member of the Communist Party of China and 0 to represent others; for family debt, we defined the family with debts as indebted families, and assigned the value of 1; defined family without debts as non-debt families, and assigned the value of 0; for the definition of urban dummy variables, we mainly referred to Lin et al. (2017).

#### Change the Measurement of the Key Variable

In order to avoid the difference in indicator definitions from affecting the empirical results, we used the “demolition area” as the proxy variable of “house demolition.” The reason for choosing the demolition area as the proxy variable is that the demolition area is usually highly correlated with the amount of house demolition, which meets the requirements of selecting the proxy variable. For the demolition area, we used the items “What is the acreage to be demolished?” in the CHFS (2011) questionnaire to measure the variable “demolition area.” For the data processing of the variable “demolition area,” we firstly assigned 0 to this variable for families that have not experienced demolition, and then added 1 to the newly obtained data and then took the natural logarithm transformation.

#### Delete the Demolition Samples Without Compensation

In real life, there may be cases that have experienced house demolition but have not received any financial compensation. In order to avoid this situation affecting the accuracy of the research results, we removed those families who have not received compensation for demolition from our research sample and conducted a robustness test on this basis.

Table 3 shows the above four independent robustness test results of the basic research part obtained by using different research methods. The results of the robustness test consistently show that (1) House demolition positively affects the overall level of family conspicuous consumption; (2) House demolition positively affects the average level of family conspicuous consumption; (3) Household wealth positively moderates the relationship between house demolition and the overall level of family conspicuous consumption; (4) Household wealth also positively moderates the relationship between house demolition and the average level of family conspicuous consumption; (5) Materialism value positively moderates the main effect between house demolition and the overall level of family conspicuous consumption; (6) Materialism value positively moderates the main effect between house demolition and the average level of family conspicuous consumption.

**Table 3:** Results of the robustness test.

**Method**	**1. Endogenous test**		**2. Modify model setting**	
**Dependent variable**	**Overall level of family conspicuous consumption**	**Average level of family conspicuous consumption**	**Overall level of family conspicuous consumption**	**Average level of family conspicuous consumption**
Model	1	2	3	4
House demolition	0.766^**^	0.261^**^	0.886^**^	0.292^**^
	(0.384)	(0.122)	(0.384)	(0.122)
House demolition × household wealth	1.434^*^	0.511^**^	1.836^**^	0.633^***^
	(0.751)	(0.239)	(0.749)	(0.238)
House demolition × materialism value	3.656^***^	1.270^***^	3.761^***^	1.294^***^
	(0.924)	(0.294)	(0.911)	(0.290)
*F* value	34.49^***^	36.31^***^	27.01^***^	27.95^***^
*R* ^2^	0.0492	0.1545	0.1532	0.1577
Observation	2,797	2,797	2,706	2,706
**Method**	**3. Change the measurement of the key variable**		**4. Delete the demolition samples without compensation**	
Model	5	6	7	8
House demolition	0.213^**^	0.069^**^	0.780^*^	0.249^*^
	(0.090)	(0.028)	(0.413)	(0.131)
House demolition × household wealth	0.370^**^	0.128^**^	2.483^***^	0.832^***^
	(0.173)	(0.055)	(0.806)	(0.256)
House demolition × materialism value	0.824^***^	0.287^***^	4.815^***^	1.649^***^
	(0.212)	(0.067)	(0.972)	(0.309)
*F* value	34.79^***^	36.58^***^	35.85^***^	37.58^***^
*R* ^2^	0.1489	0.1554	0.1549	0.1612
Observation	2,798	2,798	2,753	2,753

* * p <0.1*;

** * p <0.05*;

*** * p <0.01*.

*The values in parentheses are standard errors*.

*Due to space issues, the results of other control variables are not reported in our study. If necessary, they can be obtained from the authors*.

Through the above robustness tests, it can be considered that the research conclusions of our study are reliable. The conclusions show that house demolition not only significantly improves the overall level of family conspicuous consumption but also significantly increases the average level of family conspicuous consumption. What is more, the above main effects are moderated by the level of household wealth and materialism value.

## Study 2: The Relationship Between Conspicuous Consumption and Luxury Consumption

At present, scholars have different opinions on the relationship between conspicuous consumption and luxury consumption. We find that it can be roughly divided into two categories: The first category of view regards conspicuous consumption as luxury consumption and emphasizes that the most essential feature of conspicuous consumption is expensive. Veblen is the main representative of the first category of view, who innovatively proposed that conspicuous consumption is the exclusive consumption way of the “leisure class.” It is a way of consumption for consumers to show their economic strength to others by purchasing expensive goods and then achieve the goal of declaring, obtaining, or promoting social status (Veblen, 1899). Subsequently, many scholars put forward insights into conspicuous consumption based on Veblen's views, for example, some scholars point out that conspicuous consumption is multidimensional, and it is a way for consumers to purchase many expensive goods to achieve eye-catching behavior (Marcoux et al., 1997). Some scholars also believe that conspicuous consumption is the behavior of consumers relying on luxury spending to show their wealth and strength to society (Lancaster, 1966). On the contrary, the second category of view believes that conspicuous consumption is not completely equivalent to luxury consumption and emphasizes that conspicuous consumption of consumers not only relies on expensive goods. For example, some scholars believe that conspicuous consumption and luxury consumption are highly compatible, but the two are not completely equivalent (Frank, 1985). In certain situations, conspicuous consumption of people can rely on non-expensive commodities, such as ordinary clothes and shoes. Other scholars also suggest that the scope of conspicuous consumption is not limited to luxury goods or precious goods. Consumers can spend money to purchase high-price or low-price goods to achieve conspicuous purposes (Bagwell and Bernheim, 1996). Jin and Cui (2013) point out that conspicuous consumption has universal characteristics.

Based on the above analysis, it can be found that researchers have reached a consensus on conspicuous consumption on the following two points: first, conspicuous consumption is to meet the specific psychological needs of consumers (Sivanathan and Pettit, 2010); second, conspicuous consumption is a kind of visible consumption (Charles et al., 2009). However, scholars have not reached a consensus on the object (expensive/non-expensive) and subject (the leisure class/all classes) of conspicuous consumption. However, the two parts with controversy are exactly the focus of debate of scholars on whether conspicuous consumption is equivalent to luxury consumption. Therefore, to judge their specific relationships, it is necessary to clarify whether there are differences between the two in terms of “object” and “subject.”

The object of luxury consumption is named the luxury goods by society. Luxury goods are commodities (including services) with high demand-income elasticity; there are three most notable features as expensive and rare, exquisite quality, and hedonic experience (Lancaster, 1966). These salient features of luxury goods show that luxury consumption places a high demand on the economic foundation of consumers. This demand fundamentally determines the main scope of luxury consumption, that is, luxury consumption is more suitable for the “leisure class” with a strong economic foundation and less suitable for ordinary consumers with a weak economic foundation. It can be seen that luxury consumption relies on expensive goods in terms of objects. According to subjects, luxury consumption is more suitable for the “leisure class” and less applicable to the “non-leisure class.” But conspicuous consumption does not necessarily rely on expensive commodities, judging from the specific phenomenon of conspicuous consumption in our current social life. Consumers can achieve conspicuous purposes by spending money on high-price or low-price goods (Bagwell and Bernheim, 1996). For example, in real life, the conspicuous consumption of people can rely on non-expensive commodities, such as ordinary clothes and shoes (Frank, 1985), and for many ordinary families, their family economic foundation can bear appropriate conspicuous consumption behavior (Zhu, 2001). It suggests that, in terms of objects, conspicuous consumption can rely not only on expensive commodities but also on low-price commodities; in terms of subjects, conspicuous consumption is not only a consumption mode of the “leisure class” but also a consumption mode of “middle class” or “low class” (Charles et al., 2009).

In summary, conspicuous consumption and luxury consumption are similar in consumption motivations and patterns; while conspicuous consumption and luxury consumption are different in terms of the subject and the object of consumption. Therefore, conspicuous consumption and luxury consumption are not exactly the same. The two are both related but different.

In order to verify the above theoretical analysis results, following that of Gao et al. (2020), we used the questionnaire platform WJX.com to conduct an exploratory survey on conspicuous consumption. Its purpose was to reveal the differences between conspicuous consumption and luxury consumption by clarifying the definition of conspicuous consumption. Specifically, the interviewees were asked to fill out the following six questions: (1) What specific psychological needs do you think people want to meet when they engage in conspicuous consumption? (2) What characteristics do you think conspicuous products should have? (3) In your mind, which kind of products are conspicuous commodities? (4) From a price perspective, which do you think conspicuous products can be? (multiple choice questions) A. The price is low; B. The price is medium; C. The price is high, (5) From a morphological point of view, what kind of features do you think should be on conspicuous products? A. Visibility features (that is, features that can be directly observed by eyes); B. Invisible features (that is, features that cannot be directly observed by eyes); C. Both of the above are acceptable. (6) From the perspective of social class, which do you think is the object of conspicuous consumption? (multiple choice questions) A. Lower class B. Middle class C. Senior class; In addition, in the questionnaire, we also measured demographic characteristics and statistical variables. A total of 183 respondents participated in this survey (74 women, *M*_age_ = 29, *SD* = 7.41). The statistical results and discussion are as follows:

Respondents suggest that conspicuous consumption behaviors of people are mainly to meet the following psychological needs: “comparison,” “vanity,” “show off,” “enjoyment,” “building self-confidence,” “self-certification,” face awareness,” “self-satisfaction,” “pursue balance,” “social recognition,” and “social superiority.” The above textual information reflects that conspicuous consumption is one of the conscious behaviors of consumers, with the purpose of satisfying certain specific psychological needs through this behavior.
The respondents believe that conspicuous goods have “high prices” (68%), “high brand awareness” (42%), “unique and rare” (27%), and “strong design sense” (15%), and “weak functionality” (accounting for 5%) and other characteristics. To further clarify whether conspicuous goods must have expensive characteristics, then this study asked respondents about the price of conspicuous goods (multiple choice questions). In this issue, about 12% of the respondents believe that it can be a low-price commodity; about 26% of the respondents believe it can be a medium-price commodity, and about 96% of the respondents believe it can be a high-price commodity. Based on this information, we believe that expensive goods, such as luxury goods, can be conspicuous goods, but medium-price or low-price goods can also be conspicuous goods. The reason is that, in certain specific scenarios, consumers can publicly show others purchasing non-expensive goods, which can also meet certain specific psychological needs, for example, posting the gifts of Valentine's Day (such as flowers, chocolates, and cards) from boyfriends on Facebook or WeChat. In this scene, non-expensive flowers, chocolates, and cards are all conspicuous goods. Therefore, we suggest that conspicuous goods should not be constrained by price.
For the subjects of conspicuous consumption (multiple choice questions), about 22% of the respondents think they can be the lower class; about 69% of the respondents think they can be the middle class; about 61% of the respondents think they can be the upper class. It can be seen that conspicuous consumption is no longer an exclusive form of consumption for the “leisure class” in today's social era; it is also applicable to the non-leisure class (including the lower and middle classes). Based on the survey results of the abovementioned questions, and referring to the existing literature, we believe that conspicuous consumption mainly refers to the behavior of consumers who consciously display the purchased or consumed goods to others publicly to meet their own specific psychological needs.
When exploring what kind of products are conspicuous goods, the answers given by respondents can be roughly summarized into the following categories: “watches,” “mobile phones,” “clothes,” “shoes,” “bags,” “houses,” “vehicles,” “jewelry,” and “cosmetics.” Based on this text information, it can be seen that conspicuous goods have strong visibility characteristics.

In summary, the results of the exploratory survey confirm the results of the theoretical analysis above, that is, conspicuous consumption and luxury consumption are not completely equal. Specifically, the two have similarities in consumption motivation and consumption patterns. For example, conspicuous consumption and luxury consumption can meet certain specific psychological needs of consumers, and both meet these needs through visible consumption carriers. However, there are heterogeneous aspects in both the subject and the object of consumption. For example, conspicuous consumption is applicable to all classes and is not limited to price; while luxury consumption is more applicable to the “leisure class” and is limited to price (expensive).

## Results and Discussion

House demolition and conspicuous consumption are two topics that have been hotly discussed since the twenty-first century. We connect house demolition and conspicuous consumption for the first time in the context of the rapid advancement of the urbanization process and the increasing conspicuous consumption of China. First, we adopted the 2010 CHFS micro-survey data, took the household as our research object, and built the corresponding model setting based on the control variables on family characteristics, household head characteristics, and regional characteristics, which aim to examine the relationship between house demolition and family conspicuous consumption from the overall level and the average level. Then, in order to deepen the theoretical and practical significance of the research, we further examined the moderating effects of household wealth and materialism value on the main effect between house demolition and family conspicuous consumption. Finally, considering that the current academic research is still in the status of inadvertently clarifying the relationship between conspicuous consumption and luxury consumption, in order to further clarify the relationship between the two and deepen the understanding of the research and the public, we investigated their relationship from a theoretical perspective. And we used exploratory investigation methods to verify the results.

We have obtained three main conclusions through a series of studies. First, house demolition has a significant positive impact on family conspicuous consumption, which is not only reflected in the overall consumption level but also in the average consumption level. Second, the main effects are all positively moderated by the level of household wealth and materialism value. Third, conspicuous consumption and luxury consumption are not completely equal. They are similar in the consumption motivation and consumption pattern, but there are differences in the consumption subject and the consumption object.

This study puts forward the following practical suggestions based on the above research conclusions to improve the situation of increasing conspicuous consumption in China. First, house demolition is already an unavoidable fact in the process of rapid urbanization. When the government organizes house demolition, it should promptly transmit correct consumption concepts to demolition households to ease their conspicuous consumption motivation. For families that have completed demolition, the government also needs to pay attention to their conspicuous consumption dynamics and should provide timely advice or guidance when necessary. Second, materialism value significantly increases the main effect between house demolition and family conspicuous consumption. In this regard, the government should devote itself to cultivating residents to establish a correct world outlook and value, and dilute the desire of the residents for possession of the material. Third, conspicuous consumption is essentially an irrational consumption (Li et al., 2016). We confirmed that the level of household wealth positively moderates the relationship between house demolition and family conspicuous consumption. Therefore, the government can establish a corresponding social security system in order to prevent certain wealthy families from falling into financial distress due to excessive engagement in conspicuous consumption, which will cause a series of unnecessary social troubles.

Our study also has the following shortcomings, which are also research directions that can further explore in the future. First, through theoretical analysis, we conclude that processing fluency may be the psychological mechanism of house demolition affecting the conspicuous consumption of families. Limited to the data, we do not verify this. In future research, scholars can use other valid data to verify it and further enrich the existing research results. Second, we studied the impact of house demolition on family conspicuous consumption. Due to the particularity of the core independent variable (house demolition), we based on a micro survey database and used micro empirical methods, which are a more appropriate choice compared with choosing other research methods (such as experimental research methods). However, this choice leads to the following problems in the definition of variables: limited to research data, the definition method used in our study can effectively measure materialism value to a certain extent, but there are gaps between this method and experimental research methods to some extent. In the future, we can use unique data and adopt better definition methods to further verify the influence of materialism value on the relationship between house demolition and family conspicuous consumption.

## Data Availability Statement

The original contributions presented in the study are included in the article/supplementary material, further inquiries can be directed to the corresponding authors.

## Ethics Statement

The studies involving human participants were reviewed and approved by China National Knowledge Infrastructure (CNKI) Ethics Committee. Written informed consent to participate in this study was provided by the participants' or participants' legal guardian/next of kin.

## Author Contributions

All authors listed have made a substantial, direct and intellectual contribution to the work, and approved it for publication.

## Funding

This paper was supported in part by the National Social Science Foundation of China (No. 21CGL019).

## Conflict of Interest

The authors declare that the research was conducted in the absence of any commercial or financial relationships that could be construed as a potential conflict of interest.

## Publisher's Note

All claims expressed in this article are solely those of the authors and do not necessarily represent those of their affiliated organizations, or those of the publisher, the editors and the reviewers. Any product that may be evaluated in this article, or claim that may be made by its manufacturer, is not guaranteed or endorsed by the publisher.

## Footnotes

^1^The 27th of the regulations on “Expropriation and Compensation Ordinance of House on State-Owned Land” clearly stipulates that no unit or individual may resort to violence, threats, or illegal methods, such as interrupting water supply, heating, gas, power supply, and road traffic to force the expropriated person to relocate. Thus, house demolition in the administrative region of China is a voluntary behavior of each citizen, and is not compulsory. In the China context, house demolition is an exogenous variable.
